# Report of an unsual case of anophthalmia and craniofacial cleft in a newborn with *Toxoplasma gondii* congenital infection

**DOI:** 10.1186/s12879-017-2565-8

**Published:** 2017-07-03

**Authors:** Gabriel Emmanuel Arce-Estrada, Valeria Gómez-Toscano, Carlos Cedillo-Peláez, Ana Luisa Sesman-Bernal, Vanessa Bosch-Canto, José Luis Mayorga-Butrón, José Antonio Vargas-Villavicencio, Dolores Correa

**Affiliations:** 1 Laboratory Inmunología Experimental, Instituto Nacional de Pediatría (INP), Secretaría de Salud, Torre de Investigación, Av. Insurgentes Sur 3700-C, Col Insurgentes Cuicuilco, 04530 Ciudad de México, DF Mexico; 2 Servicio de Infectología, Instituto Nacional de Pediatría (INP), Secretaría de Salud, Ciudad de México, Mexico; 3 Servicio de Cirugía Plástica, Instituto Nacional de Pediatría (INP), Secretaría de Salud, Ciudad de México, Mexico; 4 Servicio de Oftalmología, Instituto Nacional de Pediatría (INP), Secretaría de Salud, Ciudad de México, Mexico; 5 Servicio de Otorrinolaringología, Instituto Nacional de Pediatría (INP), Secretaría de Salud, Ciudad de México, Mexico

**Keywords:** Anophthalmia, Congenital toxoplasmosis, Craniofacial cleft, *Toxoplasma gondii*, Case report

## Abstract

**Background:**

We present one unusual case of anophthalmia and craniofacial cleft, probably due to congenital toxoplasmosis only.

**Case presentation:**

A two-month-old male had a twin in utero who disappeared between the 7^th^ and the 14^th^ week of gestation. At birth, the baby presented anophthalmia and craniofacial cleft, and no sign compatible with genetic or exposition/deficiency problems, like the Wolf-Hirschhorn syndrome or maternal vitamin A deficiency. Congenital toxoplasmosis was confirmed by the presence of IgM abs and IgG neo-antibodies in western blot, as well as by real time PCR in blood. CMV infection was also discarded by PCR and IgM negative results. Structures suggestive of *T. gondii* pseudocysts were observed in a biopsy taken during the first functional/esthetic surgery.

**Conclusions:**

We conclude that this is a rare case of anophthalmia combined with craniofacial cleft due to congenital toxoplasmosis, that must be considered by physicians. This has not been reported before.

## Background

Toxoplasmosis is an infectious disease caused by *Toxoplasma gondii*, a cosmopolitan parasite [1]. The congenital form occurs when a woman is infected for the first time close to or during pregnancy [2, 3]. In early pregnancy infections, the congenitally infected fetuses commonly develop severe problems, including hydrocephalus, intraparenchymal calcifications, psychomotor retardation, seizures, hyperproteinorrachia, retinochoroiditis, uveitis, microphthalmia, cataract and, less frequently, hearing loss [2].

With the aim to study the spectrum of congenital toxoplasmosis in Mexico, we are performing prenatal and postnatal screening projects, and forming a cohort, which also includes clinical cases sent to INP for differential diagnosis and management. Among the latter, one special case claimed our attention because it was quite rare: he presented anophthalmia and craniofacial cleft, which are present very rarely together. In this article, we show this case because the evidence suggests these congenital abnormalities were due to *T. gondii* infection.

## Case presentation

**Fig. 1:**
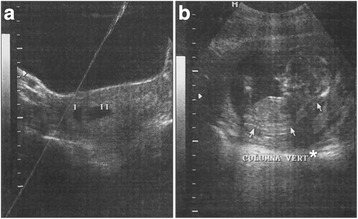
Gestational ultrasounds **a**) Ultrasound at 7.3 WG showing an intrauterine dichorionic diamniotic gestation (I, II). **b** Ultrasound at 14 WG showing involution of one of the two sacks. Arrows indicate fetal vertebral column (*)

A two-month-old male was brought to the Infectology service of INP to seek medical care, because he had dysmorphias. Also, maternal acute toxoplasmosis was suspected at six weeks of gestation (WG) due to abnormal evolution and presence of IgM anti-*T. gondii* antibodies (abs). The nineteen-year-old woman harbored intrauterine dichorionic diamniotic twins of 7.4 (gestational sac of 2.4 cm) and 7.3 (2.3 cm) WG as calculated from the ultrasound (USG) (Fig. 1a). She had miscarriage threat at 11 WG. At the 14th WG, only one apparently normal fetus was apparent in the USG, so there was involution of the other twin (Fig. 1b). A unilateral complete palatal defect was seen in the USG performed during the last trimester. A male product was born by cesarean at 38 WG; with a weight of 2.78 kg (P_10_), a height of 48 cm (P_10–25_), an Apgar score of 8/9 and a Silverman score of 0 points. The newborn lacked the left eye and presented ipsilateral oblique oro-orbital cleft. The diagnostic approach began four days after birth with a TORCH profile. IgG was positive for *T. gondii*, Rubella and Cytomegalovirus, but IgM abs were negative against the three agents. The blood count was normal. The baby presented bilateral sensorineural hearing loss as demonstrated by auditive screening. The metabolic screening results were normal.

**Fig. 2:**
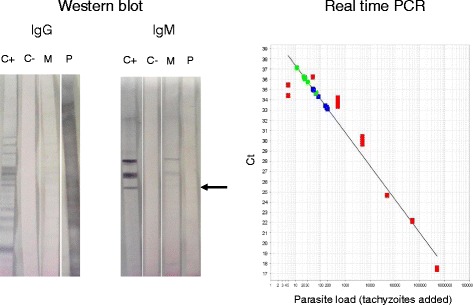
Laboratory confirmation of *T. gondii* congenital infection. Left: Western blot analysis of IgG and IgM antibody response of the patient (P) and his mother (M). C+ and C- are positive and negative newborn controls. The *arrow* indicates a slight but persistent IgM band in the baby (present in the second sample, not shown). Right: Real Time PCR quantification of the parasite load. The *blue* squares correspond to different dilutions of the maternal blood DNA, while the *green* ones are from the patient. The standard curve (*red* squares) is constructed by adding different numbers of RH strain tachyzoites to a negative blood and performing extraction and PCR procedures in parallel to those of the samples

When the patient arrived to INP, no evidence of psychomotor retardation was found and the Ophthalmology service discarded alterations in the healthy eye. Cytomegalovirus and *T. gondii* infections were searched in blood by serology and real-time PCR. Congenital toxoplasmosis was confirmed by the presence of IgM abs and IgG neoabs in western blot, as well as by real time PCR in blood (Fig. 2), with a parasite load of 6903 parasites/mL [4, 5].

**Fig. 3:**
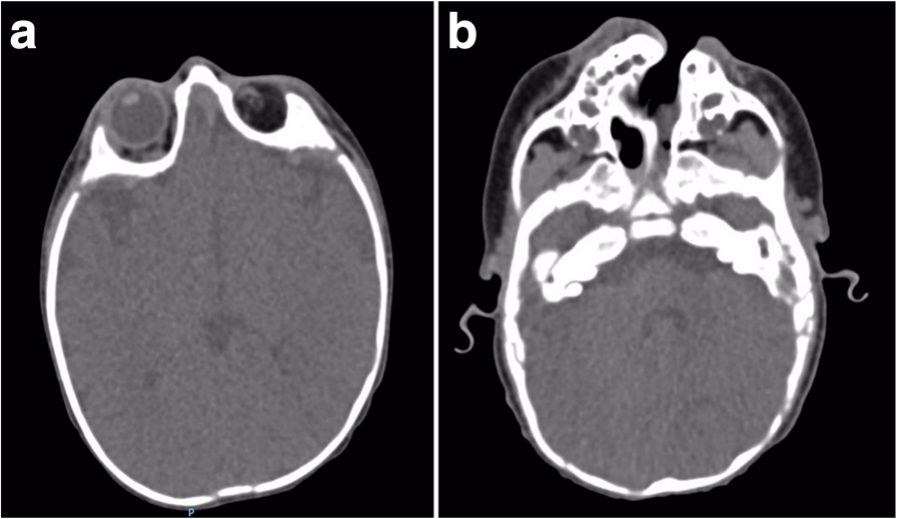
Cranial tomography findings. **a** Left anolphthalmia with hypoplastic extraocular muscles. No evidence of damage in the encephalon was seen. **b** Left oblique oro-orbitary cleft

In the cranial CT scan (Fig. 3), the left oblique oro-orbital and parasagittal cleft defect were observed, as well as ipsilateral lack of osseous tissue and soft palate. Left choanal atresia and narrow-left vestibule were discovered in the nasal cavity. The right nasal cavity was communicated with the oropharynx through a narrow choanal passage. The left turbinates were hypoplastic. A nasal endoscopy was performed which confirmed CT scan findings. The left eyeball and the optic nerve were absent; however, hypoplastic extraocular muscles were present in the orbit. The brain parenchyma and the right side of the face were normal.

According to the data described above, diagnosis was type 3 left craniofacial cleft (Tessier classification), ipsilateral anophthalmia and congenital toxoplasmosis.

Anti-*T. gondii* therapy with pyrimethamine, trimethoprim/sulfamethoxazole and folinic acid was started according to the international recommendations [2]. At the beginning, the mother did not adhere to treatment, because as she argued, there was a long distance between her home and the hospital, but mainly because she had difficulty to understand the benefits of prophylactic chemotherapy; the physicians perseverated, so the second semester of life the baby received proper treatment.

**Fig. 4:**
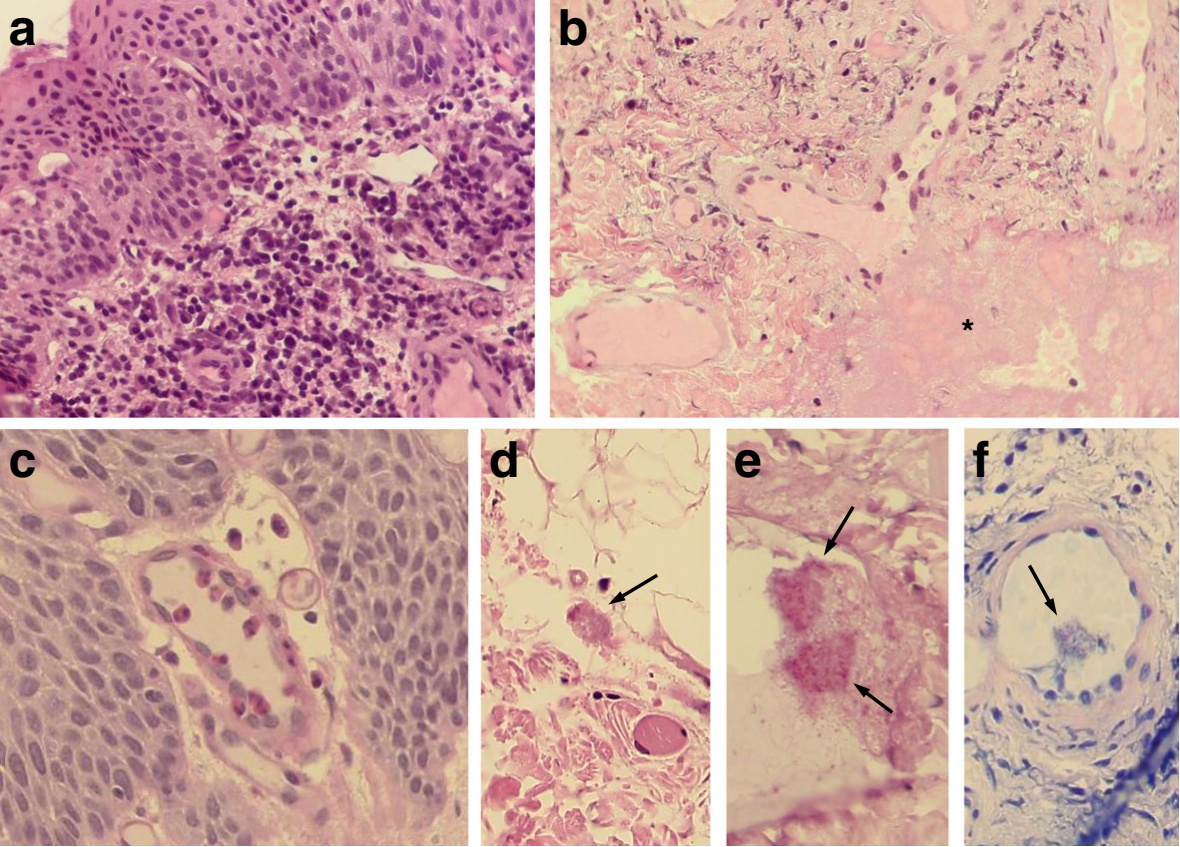
Microscopic findings of a section taken from the ocular mucosa. **a** lymphocytic infiltrate can be seen on the surface of the submucosa; **b** thrombus (*) with edema of the submucosa, degeneration of the fibers and inflammatory cell infiltrate; **c** perivascular and luminal eosinophils within epithelial blood vessel; **d** Apicomplexan pseudocyst-like structure (arrow) containing degenerated parasites; **e** and **f** PAS and Giemsa stains which show positive protozoa-like structures in the blood vessels lumen (arrows)

Surgical management was focused in maximizing actual vision and improving aesthetics, through simultaneous stimulation of soft tissues and bony orbital growth. Anophthalmia requires additional remodeling by soft tissue reconstruction and endo-orbital volume replacement (with implants, expanders and dermis-fat grafts). A first surgery was performed to start correction of the anatomical defect. During the surgery, a biopsy containing mucosa and skin was taken from the anophthalmia site. Images compatible with *T. gondii* pseudocysts and free parasites were observed by histopathology, as well as an active chronic inflammatory reaction, composed by lymphocytes, plasma cells, eosinophils and neutrophils (Fig. 4). Prognosis is favorable since the baby had no neurodevelopment disorders, no right eye or intracranial lesions and is following a protocol for reconstructive plastic surgery including eyeball prosthesis.

## Discussion

Dolk et al. [6] reported that prevalence of anophthalmia is extremely low, i.e. around 3/100,000 births. Epidemiological data suggest risk factors are maternal age over 40, multiparity, infants of low birth weight and low gestational age, none of these present in our case. There is no association to any specific race or gender. It is more commonly bilateral, while it was unilateral in the newborn shown here [7].

Anophthalmia generates early in gestation either due to failure of optic pit(s) enlargement and formation of optic vesicle(s) (*primary* anophthalmia), or to errors in anterior neural tube development (*secondary* anophthalmia). A third category (*consecutive* or *degenerative* anophthalmia) refers to cases in whom optic vesicles form, but they later degenerate and disappear. Observations of optic nerves, chiasm, and/or tracts with anophthalmia may indicate the regression of a partially developed eye [7]. We believe the present case falls into the last category, as the CT scan showed hypoplastic extraocular muscles in the orbit.

Anophthalmia can occur as a unique clinical feature, although in one-third of cases it is associated to genetic syndromes, such as the Wolf-Hirschhorn or mutations of the *SOX2* or *PAX6* genes. In these affections, although, other malformations involving the neurological and urogenital systems are usually present [7]. Genetic confirmation was not performed, mainly because he had no mental or growth retardation, abnormal size or form of the brain, or any other malformation besides craniofacial cleft, which are common alterations of the syndromes described.

Anophthalmia has also been related to environmental factors like maternal vitamin A deficiency, solvent use or exposure to X-rays, or to drugs like thalidomide, warfarin or alcohol [6]. We do not think any of these was the cause of anophthalmia, since the mother was not exposed to the referred risks and did not present clinical findings related to vitamin A deficiency along pregnancy, like blurred vision, nocturnal blindness or corneal ulcers [8]. This malformation has also been related to congenital infections, including CMV and toxoplasmosis [9, 10]. We excluded the first and found strong evidence supporting the second.

Facial cleft incidence is 1.4 to 4.9/100,000 live births [11]. Fetal craniofacial development occurs between the 3^rd^ and 8^th^ WG. It is suggested that the cleft forms when the fusion of facial processes is disrupted. However, other theories include infarction of primordial blood vessels, amniotic bands or errors in cellular migration, penetration, and differentiation [11]. The Tessier 3 cleft is the result of naso-optic groove closure failure between the frontonasal and the maxillary processes [12]. The environmental risk factors implicated in clefts are similar to those for anophthalmia, including infections [13].

Tessier did not describe anophthalmia related with type 3 cleft; this association was found later, as in our patient, but it usually includes malformations of the brain, which makes this case unique [12].

Calero-Bernal et al. [14] reported similar findings in boars caused by *T. gondii* congenital infection: the pregnant mother carried less fetuses than those expected for this species and one of them presented bilateral anophthalmia, agenesis of the nasal cartilage and prognathism. Toxoplasmosis was diagnosed by PCR and histopathology in both maternal and fetal tissues [14].

In the patient, we found active chronic inflammation in the mucosal biopsy, suggesting damage began before birth. The structures found do not have the size of bacteria nor of any blood cells, but are suggestive of *T. gondii* tachyzoites. Besides, they stained with PAS and Giemsa. The presence of thrombus and eosinophils also suggest this parasitosis as the cause of lesions [15, 16]. Since *T. gondii* is an intracellular protozoan capable of infecting any nucleated cell, and thus to interfere with any developmental process, we think congenital toxoplasmosis could be the cause of this double problem, anophthalmia and craniofacial cleft.

## Conclusion

Anophthalmia and Tessier 3 cleft are extremely rare together, especially caused by *Toxoplasma gondii* congenital infection. We report the present case for clinicians to consider this etiology when they encounter them.

## Abbreviations

abs: absorbance
CMV: Cytomegalovirus
CT: Computed Tomography (scan)
INP: Instituto Nacional de Pediatría
PAS: Periodic acid–Schiff (stain)
PCR: Polymerase Chain Reaction
TORCH: Toxoplasmosis, Rubella, Cytomegalovirus, Herpes-1/2
USG: ultrasound
WG: Weeks of Gestation

## References

[1] Weiss LM, Kim K. *Toxoplasma gondii*. The Model Apicomplexan: perspectives and methods. 2nd edition. Elsevier-Academic Press, London UK. 2014; 1085 pp.

[2] Ambroise-Thomas P, Petersen E (2000). Congenital toxoplasmosis: scientific background, clinical management and control.

[3] Dunn D, Wallon M, Peyron F, Petersen E, Peckham C, Gilbert R (1999). Mother-to-child transmission of toxoplasmosis: risk estimates for clinical counselling. Lancet.

[4] Vela-Amieva M, Cañedo-Solares I, Gutiérrez-Castrellón P, Pérez-Andrade M, González-Contreras C, Ortíz-Cortés J, Ortega-Velázquez V, Galván-Ramírez Mde L, Ruiz-García M, Saltigeral-Simentel P, Ordaz-Favila JC, Sánchez C, Correa D (2005). Short report: neonatal screening pilot study of *Toxoplasma gondii* congenital infection in Mexico. Am J Trop Med Hyg.

[5] Kompalic-Cristo A, Frotta C, Suárez-Mutis M, Fernandes O, Britto C (2007). Evaluation of a real-time PCR assay based on the repetitive B1 gene for the detection of *Toxoplasma gondii* in human peripheral blood. Parasitol Res.

[6] Dolk H, Busby A, Armstrong BG, Walls PH (1998). Geographical variation in anophthalmia and microphthalmia in England, 1988-94. BMJ.

[7] Verma AS, Fitzpatrick DR (2007). Anophthalmia and microphthalmia. Orphanet J Rare Dis.

[8] Mora JO, Gueri M, Mora OL (1998). Vitamin a deficiency in Latin America and the Caribbean: an overview. Pan Am J Publ Health.

[9] Gavinelli R (1967). On a case of congenital toxoplasmosis with agenesis of the eyeballs. Ann Ottalmol Clin Ocul.

[10] Tanferna M, Monti R (1968). Clinical considerations on a case of congenital toxoplasmosis (ocular agenesia). Minerva Ginecol.

[11] Tessier P (1976). Anatomical classification facial, cranio-facial and latero-facial clefts. J Maxillofac Surg.

[12] Zhang W, Wu H, Chen X, Li Z (2007). Tessier 3 cleft with clinical anophthalmia: two case reports and a review of the literature. The Cleft Pal-Craniofac J.

[13] Hunt JA, Hobar PC (2003). Common craniofacial anomalies: facial clefts and encephaloceles. Plast Reconstr Surg.

[14] Calero-Bernal R, Gómez-Gordo L, Saugar JM, Frontera E, Pérez-Martín JE, Reina D, Serrano FJ, Fuentes I (2013). Congenital toxoplasmosis in wild boar (*Sus scrofa*) and identification of the *Toxoplasma gondii* types involved. J Wildl Dis.

[15] Nickdel MB, Roberts F, Brombacher F, Alexander J, Roberts CW (2001). Counter-protective role for interleukin-5 during acute *Toxoplasma gondii* infection. Infect Immun.

[16] Castaño P, Fuertes M, Ferre I, Fernández M, Ferreras MC, Moreno-Gonzalo J, González-Lanza C, Katzer F, Regidor-Cerrillo J, Ortega-Mora LM, Pérez V, Benavides J (2014). Placental thrombosis in acute phase abortions during experimental *Toxoplasma gondii* infection in sheep. Vet Res.

